# Evaluating the Abnormality of Bilateral Motor Cortex Activity in Subacute Stroke Patients Executing a Unimanual Motor Task With Increasing Demand on Precision

**DOI:** 10.3389/fneur.2022.836716

**Published:** 2022-05-25

**Authors:** Kate Pirog Revill, Deborah A. Barany, Isabelle Vernon, Stephanie Rellick, Alexandra Caliban, Julie Tran, Samir R. Belagaje, Fadi Nahab, Marc W. Haut, Cathrin M. Buetefisch

**Affiliations:** ^1^Department of Psychology, Emory University, Atlanta, GA, United States; ^2^Department of Neurology, Emory University, Atlanta, GA, United States; ^3^Department of Behavioral Medicine and Psychiatry, Rockefeller Neuroscience Institute, West Virginia University, Morgantown, WV, United States; ^4^Department of Neurology, Rockefeller Neuroscience Institute, West Virginia University, Morgantown, WV, United States; ^5^Department of Radiology, Rockefeller Neuroscience Institute, West Virginia University, Morgantown, WV, United States; ^6^Department of Rehabilitation Medicine, Emory University, Atlanta, GA, United States; ^7^Department of Radiology, Emory University, Atlanta, GA, United States

**Keywords:** fMRI, stroke, motor cortex, hand function, motor recovery-cerebral infarct

## Abstract

Abnormal contralesional M1 activity is consistently reported in patients with compromised upper limb and hand function after stroke. The underlying mechanisms and functional implications of this activity are not clear, which hampers the development of treatment strategies targeting this brain area. The goal of the present study was to determine the extent to which contralesional M1 activity can be explained by the demand of a motor task, given recent evidence for increasing ipsilateral M1 activity with increasing demand in healthy age-matched controls. We hypothesized that higher activity in contralesional M1 is related to greater demand on precision in a hand motor task. fMRI data were collected from 19 patients with ischemic stroke affecting hand function in the subacute recovery phase and 31 healthy, right-handed, age-matched controls. The hand motor task was designed to parametrically modulate the demand on movement precision. Electromyography data confirmed strictly unilateral task performance by all participants. Patients showed significant impairment relative to controls in their ability to perform the task in the fMRI scanner. However, patients and controls responded similarly to an increase in demand for precision, with better performance for larger targets and poorer performance for smaller targets. Patients did not show evidence of elevated ipsilesional or contralesional M1 blood oxygenation level-dependent (BOLD) activation relative to healthy controls and mean BOLD activation levels were not elevated for patients with poorer performance relative to patients with better task performance. While both patients and healthy controls showed demand-dependent increases in BOLD activation in both ipsilesional/contralateral and contralesional/ipsilateral hemispheres, patients with stroke were less likely to show evidence of a linear relationship between the demand on precision and BOLD activation in contralesional M1 than healthy controls. Taken together, the findings suggest that task demand affects the BOLD response in contralesional M1 in patients with stroke, though perhaps less strongly than in healthy controls. This has implications for the interpretation of reported abnormal bilateral M1 activation in patients with stroke because in addition to contralesional M1 reorganization processes it could be partially related to a response to the relatively higher demand of a motor task when completed by patients rather than by healthy controls.

## Introduction

Persistent compromised hand function is recognized as one of the most common long-term deficits after stroke (1). It is well known from non–human primate studies that normal hand function relies on the integrity of the corticospinal tract (CST) that directly connects primary motor cortex (M1) neurons to spinal alpha motoneurons (2–5) and that hand function recovery after stroke relies on the anatomo-functional reorganization of viable neuronal tissue in M1 of the lesioned hemisphere (6). What is less understood is the role of the M1 of the hemisphere not injured by stroke (contralesional M1) in supporting skilled hand function. In early task-based imaging studies of patients with stroke, bilateral activation of motor areas, including the primary motor cortex (M1), has been reported when moving the affected hand (7–13). Because this was not seen in healthy controls and there was a shift toward a more normal unilateral activation pattern of ipsilesional motor areas accompanying an improvement in patient performance during the post-stroke recovery process, bilateral activation was reported as abnormal compared to healthy controls (7–13). Following up on these early findings, subsequent task-based fMRI studies demonstrated that the persistence of the bilateral activation pattern was associated with poorer motor function and recovery after stroke (14–17). This has prompted rehabilitation treatment approaches that target contralesional M1 using non–invasive neuromodulation (18).

However, the role of contralesional M1 in supporting upper limb and hand function is not clear and some seemingly contradictory results were reported with respect to neuromodulation treatment approaches targeting contralesional M1. Contralesional M1 activity appears to interfere in at least a subset of patients (19–21). In these patients, decreasing contralesional M1 excitability by cortical stimulation results in improved performance of the paretic limb (21–23). However, other reports indicate that contralesional M1 activity supports upper limb and hand function (13, 24–29) and decreasing contralesional M1 activity may result in deterioration of paretic limb performance (28, 29). There is evidence derived from rodent stroke models that reorganizational changes in contralesional M1 occur that include long-term changes in neurotransmitter systems, dendritic growth, and synapse formation (30–36). Inhibiting the contralesional hemisphere generates more behavioral deficits in the impaired forelimb in comparison to control animals, indicating that these reorganized neuronal circuits support the function of the forelimb (37).

More recent evidence of ipsilateral M1 activity in healthy subjects when executing tasks of increasing complexity or demand on precision (38–42) raises the question of whether, in addition to reported stroke-related contralesional M1 reorganization processes in pre-clinical studies (36, 43, 44), contralesional M1 activity during motor performance post-stroke could also be partially explained by task demand (13, 45). In previous imaging studies, only simple motor tasks such as hand grip or wrist movements were tested, so patients with more impaired hand function may have activated bilateral M1 in response to increased task demand compared to patients with only mild to moderate impairment. Further, in several imaging studies, mirror movements of the non-affected hand during movements of the affected hand (18) were reported. These data raise the possibility that contralesional M1 activity is partially related to mirror movements of the non-affected hand (46). As mirror movements and co-activation of the non-affected hand occur more frequently in patients with greater impairment of upper extremity function (46, 47), the presence of these movements could distort the interpretation of persistent bilateral M1 activation. Taken together, there are remaining questions about reported abnormal contralesional M1 activity in patients with compromised upper limb and hand function after stroke, which hampers the development of treatment strategies targeting this brain area.

In the present study of subacute patients with stroke, our aim was to determine the extent to which contralesional M1 activity is related to the demand on precison of a motor task and whether this activity differs between patients and healthy age-matched controls. A strong demand-dependent increase in contralesional M1 would suggest that some of the reported findings on increased contralesional M1 activity in patients with stroke could be explained by the relatively higher demand for executing a task with the impaired limb rather than reorganizational changes. We hypothesized that patients with stroke would exhibit higher activity in contralesional M1 than healthy controls and that contralesional M1 activation would depend on the demand for precision in a hand motor task.

## Materials and Methods

### Study Design

We conducted a prospective longitudinal study of patients with stroke to study motor recovery. Measures of brain structure and function, as well as hand motor function, were obtained at two-time points, in the subacute (1-month post-stroke) and the chronic stroke recovery period (6 months). In the present paper, we report the results of fMRI of the brain during a hand motor task obtained in patients with stroke during the subacute recovery period and in healthy right-handed, age-matched controls. This experiment was designed for a subset of patients with stroke with sufficient dexterity of affected hand function to execute a skilled hand motor task to determine the extent to which contralesional M1 activity is related to the demand on the precision of a hand motor task and whether it differs from a healthy age-matched control population.

### Participants

All patients with stroke referred to centers of the NIH StrokeNet of all genders, all races, and ethnicity aged 40–80 years were considered for inclusion in the main study provided they met the following inclusion criteria (1): Single lesion cerebral ischemic infarction <1 month affecting the M1 output system of the hand (M1 or CST) at a cortical or subcortical level as defined by MRI of the brain (2), paresis of the upper extremity (as assessed by NIHSS at admission or chart review) (3), no other neurological disorders (4), no aphasia that prevented participants from following instructions or from communicating effectively with the study team, (5) no dementia (see below for details) (6), no or only mild depression [Hamilton Depression score of ≤ 13 (48)] (7) no contraindication to transcranial magnetic stimulation or MRI (8), no intake of CNS active drugs that block plasticity (for example benzodiazepines) (9), the ability to give informed consent. For participation in the fMRI study, patients had to meet additional inclusion criteria with respect to their level of affected hand function. Participants had to be able to manipulate a joystick with the affected hand with sufficient precision to achieve a minimum accuracy standard on the hand motor task (see below for details). Hand function was assessed using the Jebsen Hand Function Test (JHFT), a standardized test of hand function (49). The raw score was normalized to age- and sex-matched standard scores that accounted for hand dominance (49, 50). A normalized score greater than zero indicated abnormal hand function, with higher values indicating more severe impairment.

Healthy control participants fulfilled the same criteria for participation in the present study (age between 40 and 80 years; no neurological or psychiatric disorders; no current usage of central nervous system active drugs; no contraindication for MRI; normal cognitive function as evaluated by the RBANS) except for the criteria pertaining to the stroke, as all had normal neurological examinations and normal brains as evaluated by MRI. In addition, all control participants were strongly right-handed as confirmed by the Edinburgh Handedness Inventory (51).

All procedures involving human participants were approved by the Institutional Review Board at Emory University. Procedures followed were in accordance with the ethical standards of the Institutional Research Committee on Human Experimentation (institutional and national) and with the Helsinki Declaration of 1975, as revised in 2008. All participants gave written informed consent and were blinded to the stated hypothesis of the experiments.

### MRI Acquisition

Structural and functional MRI scans of all participants were collected on a Siemens Prisma 3T MRI system with a 64-channel head coil. T1-weighted structural scans were collected according to the HCP Lifespan Project (52, 53) MPRAGE protocol: TR = 2,400 ms, TE = 2.24 ms, flip angle = 8 degrees, FOV = 256 x 240 x 208 mm, voxel size 0.8 mm^3^. In a separate scanning session, task-based functional MRI runs were collected with the following protocol: TR = 2,000 ms, TE = 28 ms, flip angle = 90 degrees, FOV = 192 x 192 x 102 mm, voxel size = 3 mm^3^, 163 volumes per run. Patients with stroke completed four task runs (total duration 22 min) using the affected hand only, while healthy controls were able to tolerate a longer scanning session without fatigue and completed eight task runs, four with the dominant right and four with the non–dominant left hand (total duration 44 min). These scan parameters were also used to acquire data from a separate motor localizer task to independently define the M1 region of interest (ROI) used in all analyses. Participants repeatedly opened and closed either their right or left hand at approximately 1 Hz in response to a visual cue. Twenty movement blocks of 10 s (10 per hand) alternated with 10 resting blocks of 10 s (165 functional volumes, total duration 5 min and 30 s). An arrow or rest cue on the visual display specified which hand was to perform the action in each block.

### fMRI Hand Motor Task

**Figure 1:**
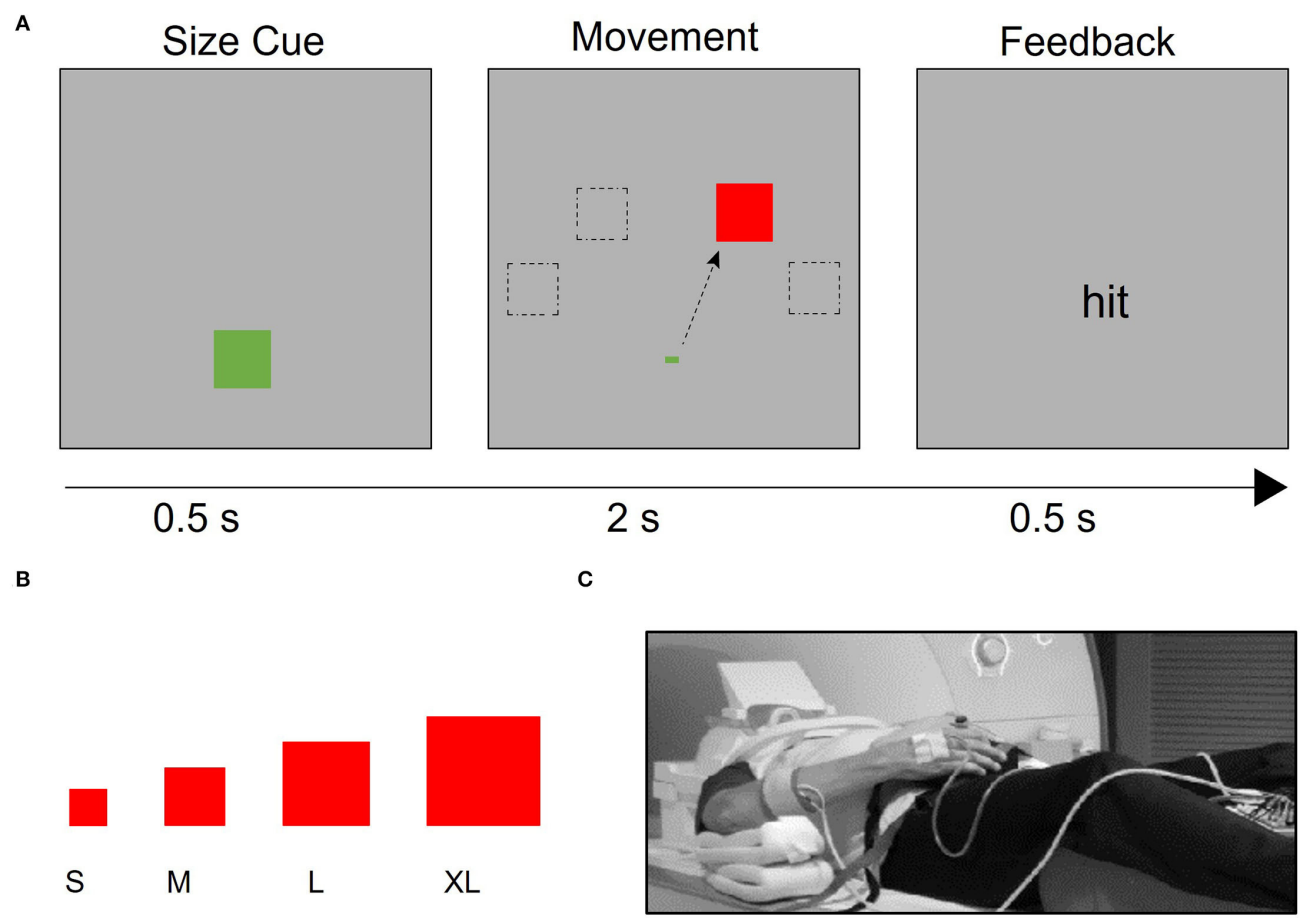
Task design. **(A)** After a target size cue, the target appears in one of four possible locations (−60°, −30°, 30°, and 60° relative to the vertical meridian). Participants were instructed to use the joystick to move the cursor to the target as quickly and accurately as possible. If the cursor began at the starting point and was inside the target 2 s after the target presentation, participants received “hit” feedback; otherwise, they received “miss” feedback. **(B)** Four different target sizes were used to vary the demand on precision, **(C)** Participants used an MRI-compatible joystick to perform the task. The joystick was secured to their body with straps and pads were placed to support the arm and minimize elbow and shoulder movement. EMG electrodes were placed on participants' right- and left- extensor carpi ulnaris (ECU) muscles, which support the manipulation of the joystick.

The hand motor task used during fMRI data collection allows for parametric manipulation of the demand on the precision of a hand movement by requiring participants to use a joystick to move a cursor into targets of varying sizes (13, 40, 41, 54) (Figures 1A,B). The use of the term ‘demand' refers to the demand on movement precision. Stimuli were projected onto a screen that was viewed *via* a mirror mounted onto the head coil using Presentation® software (www.neurobs.com). With the participant in a supine position on the scanner bed, the base of the joystick base was strapped to the torso of the participant with Velcro straps (Figure 1C). It was positioned in such a way that the participant could rest the wrist on the base of the joystick comfortably and could manipulate the joystick without moving the distal or proximal arm. Foam pads supported the arm so that participants did not have to use muscular effort to actively maintain the position of the arm or the hand on the base of the joystick. Movement epochs (16 s x 12) alternated with resting epochs (9 s x 11). Trials were blocked by target size, with four trials per movement epoch. Each target size block was presented three times per run. The order of target size blocks was randomized within each run. Patients with stroke completed four runs with the affected hand. Healthy control participants completed four runs with one hand (right or left) and then four runs with the other hand, in a counterbalanced order across participants. A trial was counted as an accurate “hit” if the midpoint of the cursor was within the boundaries of the target square at the end of the 2 s trial. Movement time was defined as the time between the onset of the target and the time at which the cursor reached and remained within the target square.

At least one day prior to scanning, participants completed a training session to familiarize themselves with the pointing task, ensure that patients had enough hand function to perform the task at a minimum level of accuracy, and minimize learning effects during the fMRI experiment. This training procedure was used to account for the differences in the level of baseline performance across participants (54). Control participants performed up to three training runs of three blocks each (seven trials of the same target size per block) with both their right and left hands. Participants completed fewer than three training runs if they had > 50% accuracy on the smallest target size at the end of a run or had improved to >50% accuracy on the next largest target size that was below 50% accuracy after the first run. Patients with stroke performed the task with the affected hand and were required to achieve a minimum accuracy of 50% on the largest target size to participate in the fMRI scanning session.

### EMG Recording and Data Analysis

The objective of the EMG analysis was to determine whether the task in the scanner was done strictly unimanually (13, 40, 41). Continuous EMG was recorded with the BrainAmp MR plus system (Brain Vision, LLC, Morrisville, NC, USA) from both extensor carpi ulnaris (ECU) muscles in the scanner, using MRI-compatible surface electrodes (Brain Vision, LLC, Morrisville, NC) placed 2 cm apart in a belly tendon montage. The ECU muscle was recorded because manipulating the joystick involved extensions and flexions of the hand (40, 41). EMG signals were band-pass filtered (5 Hz−1 kHz), amplified, digitized, sampled at a 5,000 Hz frequency, and stored for offline analysis. EMG data for the non–performing hand were first corrected for MR artifacts in BrainVision Analyzer 2 (version 2.1.2, Brain Products GmbH, Gilching, Germany) using the artifact subtraction method (55) with template drift detection and a sliding average calculation of 11 TR intervals (22 s). Next, data were downsampled to 1,000 Hz, baseline-corrected, rectified, and segmented into the task (16 s) and rest (9 s) blocks. Following Barany et al. (41), mean EMG activity for the non–performing hand was compared for task and rest blocks. EMG activity of the performing hand was not quantified due to the additional MR artifacts induced by large movements. The absence of task-related changes in the non-performing hand activity was assumed when the mean EMG amplitude of the ECU muscle of the non-performing hand during movement blocks was similar to that in resting blocks (13, 40, 41). For healthy control participants, a repeated-measures ANOVA with task period (movement/rest) and non-performing hand (right/left) as factors were used to determine whether performing hand activity (i.e., during the task period or during the rest period) was related to changes in mean EMG amplitude of the non-performing hand. As the patients with stroke only performed the task with the affected hand, a paired *t*-*test* was used to evaluate whether EMG activity for the non-performing hand differed between the active and rest blocks.

### MRI Data Analysis

All processing of the fMRI data used Analysis of Functional NeuroImages [AFNI; (56)]. fMRI preprocessing steps included slice time correction, head motion correction, 12-parameter affine alignment between the structural and functional images, and non-linear warping between the structural image and the MNI152 2009 template in MNI space, smoothing with an FWHM 6 mm smoothing kernel, and conversion to percent signal change. As the brains of individuals with stroke are more difficult to normalize, we used enantiomorphic normalization (57) to transform the patient brains, with the transformation from non-linear warping of the “healed” structural image applied to the original lesioned brain image. The transformations for head motion correction, coregistration, and normalization were concatenated and applied in a single step to the functional data before smoothing to reduce the number of interpolation steps. Following preprocessing, a general linear model (GLM) analysis was performed using AFNI's 3d Deconvolve tool. Separate regressors were created for each combination of target size and hand, with block time (16 s) convolved with the GAM function. In addition to these task regressors, six head movement vectors were included as regressors of no interest. Volumes with more than 0.9 mm total head movement were censored.

**Figure 2:**
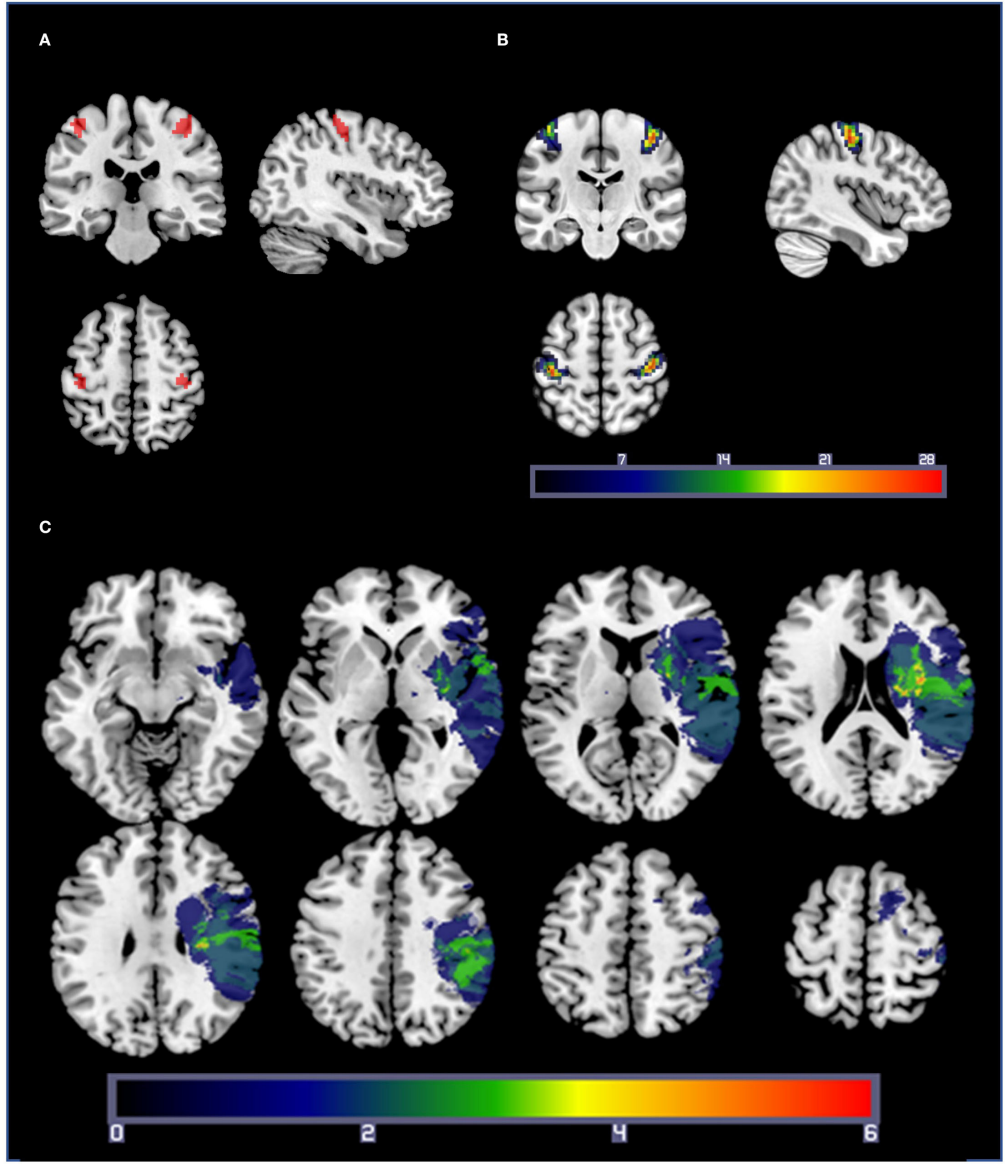
**(A)** A representative set of M1 ROIs from a healthy control participant, based on the comparisons of right-hand movement > rest and left-hand movement > rest from the hand localizer scan. Statistical thresholds were set on an individual basis to identify a cluster of 50 contiguous voxels centered in M1 for each participant. **(B)** ROI overlap map: Summation of M1 ROIs for each participant (healthy controls and patients with stroke). Warmer colors indicate a voxel is included in more ROIs. ROIs were densely clustered over the posterior aspect of the precentral gyrus including the hand knob and the central sulcus. **(C)** Lesion overlap map: lesion masks from the 19 patients with stroke projected into standard space. Lesions from the right hemisphere were flipped across the sagittal axis for illustration purposes only. Warmer colors indicate a voxel is present in more participants' lesion masks.

Hand motor localizer task data were analyzed separately, with regressors for blocks of right- and left-hand movement. For each participant, functional ROIs were created by selecting a threshold (mean ± SD; *t* = 10.5 ± 4.2) at which a cluster of 50 contiguous voxels centered in anatomical M1 could be found for the comparisons of left hand > rest and right hand > rest. For patients with stroke, any voxels intersecting the lesion mask of the individual were subtracted from the ROI. Due to the contiguity requirement, clusters with exactly 50 voxels could not be found for 19 of the 100 ROIs, so the cluster size closest to 50 voxels was used (mean ± SD cluster size: 50.2 ± 1.9 voxels). These ROIs were then used to extract beta values for each combination of target size and hand for the fMRI joystick pointing task. A representative sample ROI for one participant is shown in Figure 2A. As seen in Figure 2B, ROI location varied from participant to participant but was densely clustered over the posterior aspect of the precentral gyrus including the hand knob and the central sulcus. For three patients with stroke, the hand localizer task did not generate reliable clusters of 50 voxels that rose above the level of general noise in one or both hemispheres. For these three patients, we substituted a 50-voxel ROI generated from a group-level analysis of the healthy control participants' hand localizer data, with any intersections with the lesion mask subtracted out. The results reported below do not differ if the data from these three participants are excluded.

### Statistical Analysis

In our previous work (40, 41), we have used repeated-measures ANOVAs to look for group-level effects of target size on brain activity or on certain aspects of behavioral performance like accuracy or movement time. However, this approach only indicates that target sizes are responded to differently but does not require activation changes to be ordered or linear and fails to capture individual differences in how target size may affect behavioral performance or brain activation. Thus, we additionally find a best-fitting line for the four target size data points (for either task accuracy or BOLD activation) for each participant and take the slope of that line as a measure of the demand on the precision of the motor task, henceforth the *demand function* for each participant; essentially, a measure of how much the behavior of an individual or brain activation changes linearly as the demand on movement precision increases.

## Results

### Participants

In total, 19 patients (12 F, mean age ± SD: 59.4 ± 9.9, range 40–76) and 31 healthy, right-handed, age-matched controls (17 F, age: 61.7 ± 9, range 50–78) participated in the study. The mean age of the two groups did not differ [*t*(48) =0.84, *p* =0.4]. Data from a subset of the healthy controls were reported in a previous paper (41). Demographic information, including neuropsychological testing and stroke-specific information, is summarized in Table 1. A lesion overlap mask for the 19 patients with stroke is in Figure 2C. Half of the patients had a dominant affected hand, and five patients were left-handed (see Table 1 for details). The Repeatable Battery for the Assessment of Neuropsychological Status (RBANS) (58) was used to screen for cognitive impairment. Any patient scoring 2 SD below the mean (Total Index Score <70) was clinically assessed for impairment that would indicate dementia and to determine whether they had the cognitive ability to consent and follow task instructions. Except for two patients with stroke, total index scores of all participants were above 70 (see Table 1). Two patients with stroke scored below 70 but had no clinical signs of dementia. As a group, the healthy control participants had significantly higher RBANS scores than the patient group [*t* (48) = 3.23, *p* < 0.01].

**Table 1:** Participant characteristics.

	**Patients with stroke**	**Healthy controls**
Group size	19	31
Age	59.4 (9.9)	61.7 (9.0)
Sex	12 F / 7 M	17 F / 14 M
Race	1 Asian	1 Asian
	9 Black	10 Black
	9 White	18 White
		2 DNR
Dominant hand	14 R / 5 L	31 R / 0 L
Affected hand	8 R / 11 L	
Concordance	9 concordant	
	10 discordant	
Time since stroke (days)	18.9 (7.3)	
JHFT	0.21 (0.19)	
WMFT-T (s)	2.49 (1.48)	
WMFT-GS (kg)	25.8 (13.8)	
HD	2.37 (1.98)	1.60 (2.44)
RBANS	91.9 (18.1)	105.8 (12.4)

*The format is mean (SD). DNR, did not respond; JHFT, Jebsen Hand Function Test; JHFT scores are normed for age, sex, and handedness. WMFT, Wolf Motor Function Test (mean time = T, grip strength = GS). For Hamilton Depression (HD) score, one healthy participant's data were incomplete for more than 1 item and were excluded from this analysis. In participants with single items missing, the highest score for that item was used. RBANS Total Index Mean = 100 +/– 15. The two groups did not differ on age [t(48) = 0.85, p > 0.2] or depression score [t(48) = 1.16, p > 0.2] score, but the control participants had significantly higher scores on the RBANS than the patients [t(48) = 3.23, p < 0.01]*.

### Hand Motor Task Behavior

**Figure 3:**
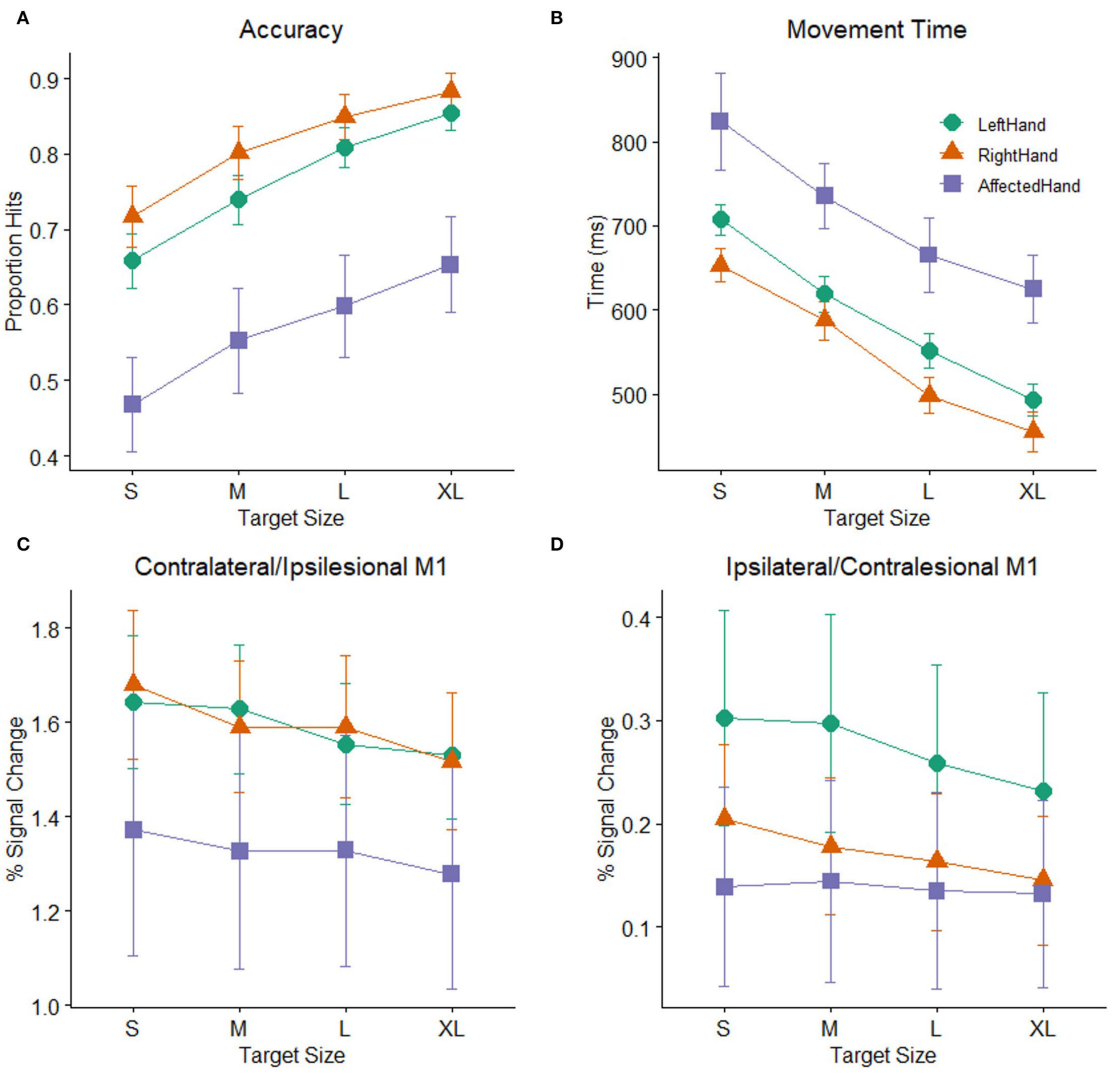
Task performance: **(A)** Accuracy (proportion hits) for each combination of hand (affected hand in patients with stroke, left hand and right hand in healthy control participants) and target size [small (S), medium (M), large (L), extra-large (XL)] during the hand motor task. Error bars represent SEM. **(B)** Mean time (in ms) from the onset of the target to when the cursor hit and remained on the target for each combination of hand and target size. Consistent with Fitts' law, participants showed greater accuracy and faster movement times as the target size increased. **(C)** BOLD response (percent signal change) during movement to targets of different sizes plotted for the contralateral M1 ROI (healthy controls) or ipsilesional M1 ROI (patients with stroke). **(D)** BOLD response (percent signal change) during movement to targets of different sizes plotted for the ipsilateral M1 ROI (healthy controls) or contralesional M1 ROI (patients with stroke).

As a group, the patients with stroke showed significant impairment on the pointing task relative to the healthy control participants (Figures 3A,B). A mixed-model ANOVA with target size as a within-subject factor and group (stroke: affected hand / control: non–dominant hand) as a between-subject factor revealed significant main effects of group [*F* (1, 48) = 10.22, *p* =0.002] and target size [*F* (2.47,118.33) = 72.54, *p* < 0.001] on accuracy, but no interaction of group and target size [*F* (2.47,118.33) =0.31, *p* > 0.2]. Patients with poorer hand function (as measured by the JHFT) tended to have lower overall task accuracy than patients with better hand function, indicated by a trend relating JHFT score and overall task accuracy (*R*^2^ =0.167, *p* = 0.08). A separate mixed-model ANOVA with target size as a within-subject factor and group as a between-subject factor showed the same pattern of effects on movement time as accuracy, with significant effects of group [*F*(1, 48) = 10.14, *p* = 0.003] and target size [*F*(1.73,82.8) = 47.67, *p* < 0.001] but no interaction of group and target size [*F*(1.73,82.8) =0.1, *p* > 0.2]. For simplicity, we report only the comparisons between the affected hand of the patients with stroke and the non–dominant (left) hand of the control participants, but the results are the same when comparing affected hand performance against dominant (right) hand performance for the controls.

Patients with stroke showed significantly lower accuracy and longer movement times than healthy controls. Nevertheless, accuracy and movement time were clearly demand-dependent in both patients and control participants. There were significant main effects of target size in both the accuracy and movement time analyses reported above. The mean demand slope measures were significantly greater than zero in all cases [control left hand: *t* (30) = 9.3, *p* < 0.001; control right hand *t* (30) = 7.01, *p* < 0.001; stroke-affected hand *t* (18) = 9.21, *p* < 0.001), indicating that accuracy decreases and movement time increases as the demand on precision increases, consistent with Fitts' Law (59, 60). The presence of this relationship in the patients with stroke indicates that their behavior followed this basic principle and supports the notion that patients and healthy participants demonstrated comparable effort despite the differences in overall accuracy and movement time.

### Overall M1 ROI Activation

While performance of patients on the pointing task was slower and less accurate than controls, mean activation in either the ipsilesional or contralesional M1 ROI in the stroke patient sample was not significantly different than mean activation in the corresponding contralateral or ipsilateral M1 ROIs of the healthy control participants. Two mixed model ANOVAs with target size as a within-subject factor and group (stroke: affected hand/ control: non–dominant hand) as a between-subject factor showed significant main effects of target size in both M1 ROIs (ipsilesional/contralateral: *F* (3,144) = 10.58, *p* < 0.001, contralesional/ipsilateral: *F*(3,144) = 2.85, *p* =0.04), but there were no main effects of group (ipsilesional/contralateral: *F* (1,48) = 1.01, *p* >0.2, contralesional/ipsilateral: *F* (1,48) =0.85, *p* >0.2) or interactions between group and target size (ipsilesional/contralateral: *F* (3,144) = 1.35, *p* >0.2, contralesional/ipsilateral: *F*(3,144) = 1.67, *p* =0.18), indicating that patients with stroke and healthy controls did not significantly differ in mean BOLD activation or in how the BOLD response was affected by target size (Figures 3C,D). We report the comparison against the healthy control non-dominant (left) hand, but the results do not differ if healthy control data from dominant (right) hand task performance is used. Neither patients with stroke nor controls showed a significant correlation between average task accuracy and average ROI BOLD activation, in either the contralateral/ipsilesional M1 ROI (stroke affected hand: *R*^2^ = 0.019, control right hand: *R*^2^ = 0.002, control left hand: *R*^2^ = 0.056, all *p* > 0.2) or the ipsilateral/contralesional M1 ROI (stroke affected hand: R^2^ = 0.032, control right hand: *R*^2^ = 0, control left hand: *R*^2^ = 0.023, all *p* > 0.2), indicating that participants with better (or worse) task accuracy do not necessarily have higher (or lower) levels of M1 activation.

### Demand-Dependent M1 Activation

As shown above, the main effect of target size indicates that participants responded differently to differently sized targets in both the contralesional/ipsilateral and ipsilesional/contralateral M1 ROIs. To determine whether patients with stroke and controls showed evidence of a linear relationship between BOLD activation and task demand in the ipsilesional/contralateral M1 ROI, we calculated the slope of the line best fitting the BOLD activation data for the four different target sizes for each participant. For both healthy controls and patients, these demand-dependent slopes were significantly non-zero in the ipsilesional/contralateral M1 ROI [control left hand: *t* (30) = −5.5*, p* < 0.001; control right hand *t* (30) = −5.56, *p* < 0.001; affected hand *t* (18) = −2.51, *p* = 0.022] with increasing BOLD activation as target size gets smaller and demand on precision increases. Demand-dependent slopes did not differ between patients and controls [*F* (1,48) = 1.12, *p* > 0.2]. In contrast, in contralesional/ipsilateral M1, only healthy control participants showed significantly non–zero demand slopes for the ipsilateral M1 ROI [control left hand: *t* (20) = −3.08, *p* = 0.004; control right hand *t* (30) = −2.57, *p* = 0.015]. For the patients with stroke, the demand slopes were not significantly different from zero [*t* (18) = −0.56, *p* > 0.2] in the corresponding contralesional M1 ROI (Figure 3D). Furthermore, an inspection of Figure 3D suggests that the slopes are different between patients with stroke and controls, with an almost flat line across target sizes in the patients. However, this difference did not reach statistical significance [*F* (1,48) = 3.97, *p* = 0.052].

**Figure 4:**
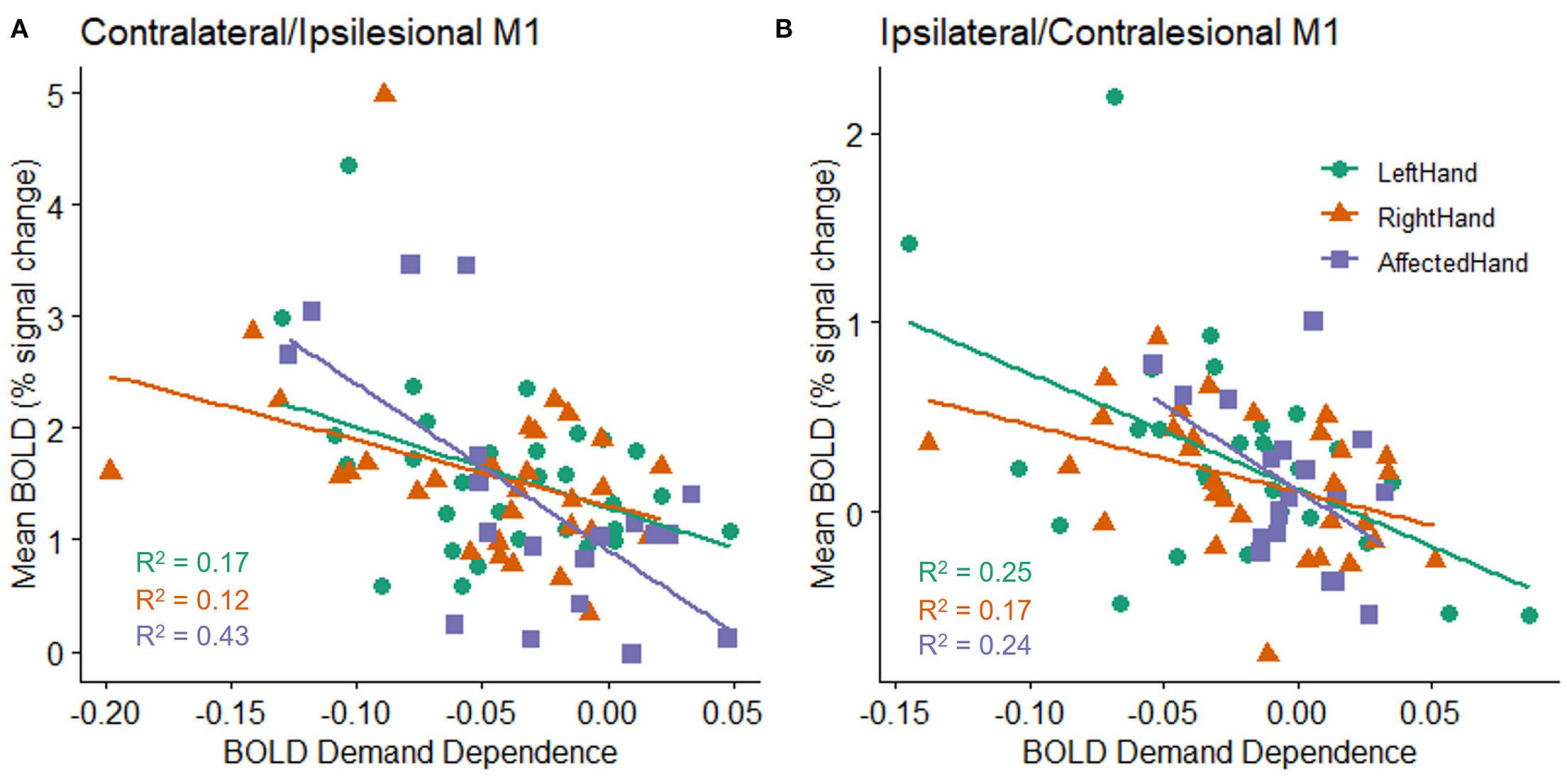
Relationship between an individual's mean BOLD response (percent signal change) across all target sizes and the slope fit to an individual's BOLD response (BOLD demand dependence) during movement to each of the four different target sizes in the **(A)** contralateral (healthy subjects) or ipsilesional (patients with stroke) M1 ROI, and **(B)** ipsilateral (healthy controls) or contralesional (patients with stroke) M1 ROI. All groups show a statistically reliable correlation between BOLD activity and BOLD demand dependence, with higher mean BOLD activation related to a steeper slope in M1 activation changes (i.e., a larger decrease in activation for the largest targets relative to the smallest targets). A negative demand dependence slope indicates less activation for large targets than small targets.

As reported above, an individual's mean BOLD activation did not correlate with their mean accuracy on the task. However, there was a relationship between the overall level of BOLD activation in an individual's M1 ROI and the likelihood of observing a non-zero slope in the BOLD response as the target size changed. This was true for both hemispheres and for both patients with stroke and healthy controls (stroke ipsilesional *R*^2^ = 0.432, *p* = 0.002; healthy right contralateral *R*^2^ = 0.123, *p* = 0.052; healthy left contralateral *R*^2^ = 0.165, *p* = 0.023; stroke contralesional *R*^2^ = 0.236, *p* = 0.035; healthy right ipsilateral *R*^2^ = 0.17, *p* = 0.021; healthy left ipsilateral *R*^2^ = 0.252, *p* = 0.004). Specifically, individuals with higher M1 ROI activation (in either hemisphere) tended to have larger changes in BOLD activity across target sizes in that hemisphere (Figure 4).

### Exploratory Analysis: Handedness

**Figure 5:**
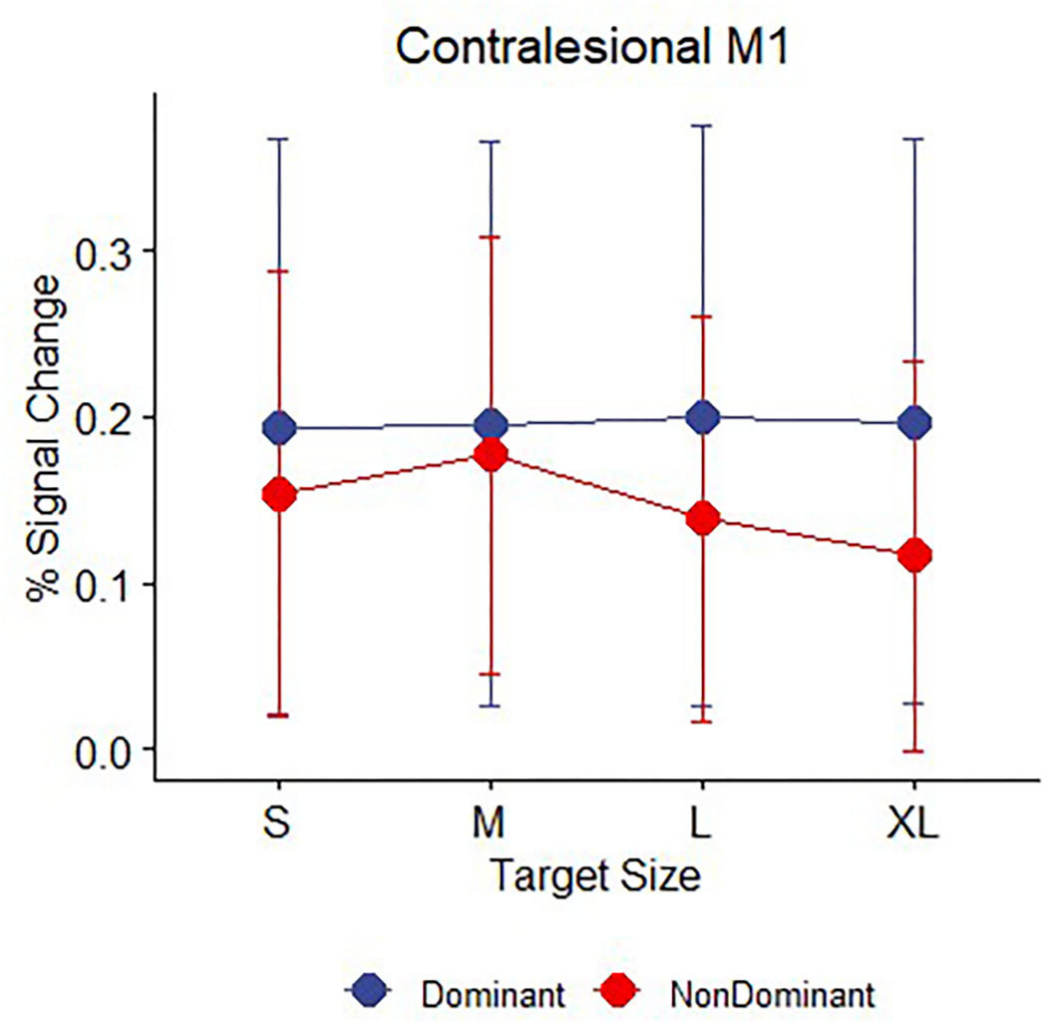
Contralesional M1 mean BOLD response (percent signal change) during movement to targets for n = 16 patients with stroke divided by whether the affected hand was dominant (blue, *n* = 8) or non-dominant (red, *n* = 8) prior to their stroke. Error bars represent SEM.

Because we have previously reported subtle differences in demand-dependent activity in ipsilateral M1 depending on whether the dominant right hand or non-dominant left hand was used in right-handed healthy controls (41), we explored the factor of handedness in our patients with stroke. Our sample of patients with stroke performed the hand motor task only with the affected hand, but of the patients who had individually localizable M1 hand ROIs (see *MRI Data Analysis*), the (previously) dominant hand was affected in eight patients and the non–dominant hand was affected in eight patients (see Table 1 for details about left and right-handedness). We examined whether there was evidence for demand dependence in contralesional M1 when the dominant or non-dominant hand was used, though caution in interpreting these results is warranted due to the very small sample sizes. Average task performance did not differ between the two groups [mean dominant = 0.542, mean non-dominant = 0.609, *t* (14) = 0.48, *p* > 0.2]. Patients who performed the task with an affected non-dominant hand showed evidence of increasing contralesional M1 activation with increasing task demand, with non-zero activation slopes [mean slope = −0.015, *t* (7) = 1.9, *p* = 0.05] indicating demand dependency, but patients who were tested using their affected dominant hand did not [mean slope = 0.001, *t* (7) = 0.24, *p* > 0.2] (Figure 5).

### Exploratory Analysis: Cognition

The JHFT used here to measure impairment of hand function in the patients with stroke is a timed test and may therefore be subject to motivational or cognitive constraints. In our patient sample, scores on the JHFT were significantly related to cognitive function as assessed by the RBANS; patients with higher RBANS scores showed significantly less impairment on the JHFT (*R*^2^ = 0.46, *p* = 0.002). However, the pointing task used here is also time-limited and could be subject to similar motivational or cognitive constraints. Indeed, across all patient and control participants, individuals with higher RBANS scores performed better on the pointing task (*R*^2^ = 0.26, *p* < 0.001); this trend was present but weaker in just the patient sample (*R*^2^ =0.16, *p* = 0.09).

### EMG Activity of the Non-performing Hand

**Figure 6:**
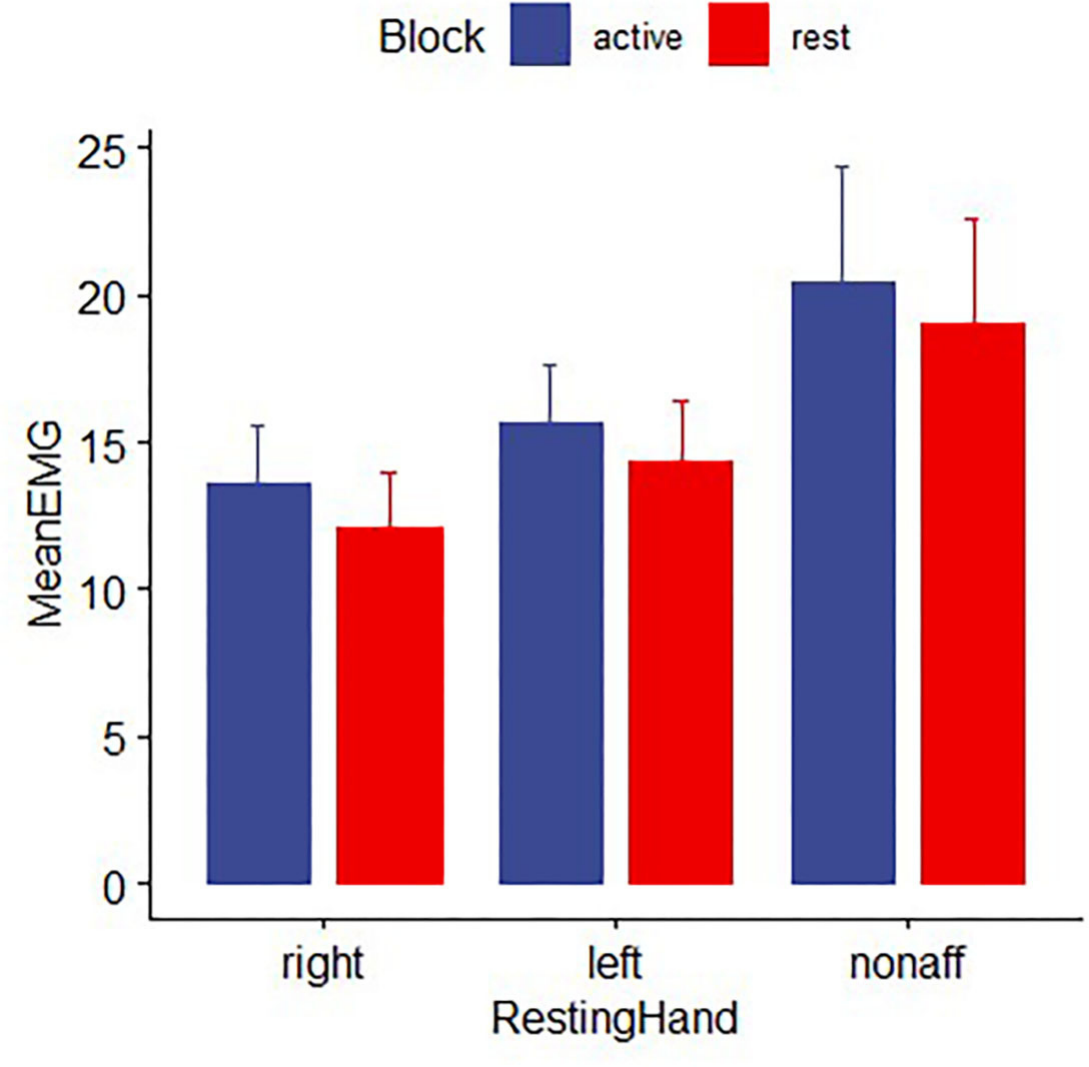
EMG activity for healthy controls and patients with stroke for the non-performing hand during time periods while the other hand was moving the joystick for the pointing task (active block) or resting (rest block). EMG in the nonperforming hand did not differ significantly between movement and rest blocks, indicating the absence of mirror movements. Error bars reflect SEM.

Electromyography data from two patients with stroke and six healthy participants were excluded from further analysis due to missing data or excessive artifacts. Both healthy control and stroke participants were able to perform the hand motor task unimanually, as mean EMG activity for the non–performing hand did not differ between movement and rest blocks for either group [controls: *F* (1,92) = 0.48, *p* > 0.2; patients: *t* (16) =0.93, *p* > 0.2; Figure 6].

## Discussion

The goal of the present study was to determine the extent to which, in patients with stroke and mild to moderate impairment of hand function due to injury of M1 or CST, activation of contralesional M1 can be explained by the demand of a motor task. While patients showed significant impairment relative to controls in their ability to perform the hand motor task in the fMRI scanner, both patients and healthy controls showed demand-dependent task performance with higher accuracy and faster movements for larger targets than smaller targets. The demand for the precision of the motor task affected the BOLD response for both M1 ROIs in patients and in healthy controls, indicating that targets of different sizes were responded to differently. However, while in healthy controls, this differential response took the form of a linear relationship between M1 BOLD activation and demand on precision in both contralateral and ipsilateral hemispheres, a similar demand-dependent profile was only seen in the ipsilesional M1 in patients with stroke, suggesting a weaker relationship between demand dependency and BOLD activation in contralesional M1. Furthermore, neither ipsilesional nor contralesional M1 showed evidence of elevated BOLD activation relative to healthy age-matched controls when executing a strictly EMG confirmed, a unimanual task with the affected hand.

Because the EMG recordings of the non-performing hand during the scanning procedures did not show any task-related changes in the magnitude of EMG activity, it is very unlikely that the BOLD response in contralesional M1 is due to mirror movements. Instead, the results of the EMG recordings demonstrate strictly unimanual execution of the task with the affected hand. Our findings confirm earlier reports of contralesional M1 activity in patients with subacute stroke involving M1 or its CST projections and mild to moderate impairment of hand function (13). The task from this early study consisted of a non-sequential finger sequence and was not designed to quantify the kinematic details that allow the parametric increase in demand on precision. In contrast to the present study, healthy age-matched controls did not show corresponding ipsilateral M1 activity when executing the non-sequential finger movement task. While we do not know the kinematic details of that task, the lack of observable activation in the ipsilateral M1 in healthy participants in that study is likely due to the relatively lower task demand (40, 41).

Because the motor task employed in the present study keeps important kinematic variables (e.g., force, amplitude, and frequency) and the muscles supporting the motor task similar across different levels of demand, the weaker relationship between the contralesional M1 BOLD response to demand on precision is likely not due to differences in the execution of the task. This notion is supported by the similarity in the demand slopes, indicating that the patients and controls responded similarly to an increase in demand on precision, with better (faster and more accurate) performance for larger targets and poorer performance for smaller targets. However, while important kinematic variables for this task were matched and both groups showed similar demand-dependent performance, patients also had significantly worse accuracy and slower movement times than controls. This raises the possibility that differences between the groups may arise due to these performance differences rather than differing neural responses to the task. Task performance was also related to cognitive function, which also differed between the groups. However, if these differences had an impact on the BOLD activity, we would expect an increase in BOLD activity in patients relative to controls since patients found the task more difficult, with longer movement times and lower accuracy. Instead, we see reduced or similar activation in the patient group. Therefore, differences in M1 activation between patients and controls cannot be explained by differences in task performance or cognitive function.

We do not have direct data on the vasculature in our participants and the patients likely have an arteriosclerotic disease that may compromise their hemodynamic response function. However, there is no statistically significant difference between the overall BOLD activation in contralesional M1 compared to the healthy control ipsilateral M1 or the ipsilesional M1 compared to healthy control contralateral M1. Further, we would not expect compromised hemodynamic response function to have a systematic impact on one hemisphere (i.e., contralesional M1) compared to the other hemisphere (i.e., ipsilesional M1) or for this to explain the weaker relationship between M1 BOLD activation and demand on precision in contralesional M1.

Compared to the mean ipsilesional/contralateral M1 BOLD response, the mean contralesional/ipsilateral M1 ROI BOLD response is substantially smaller. While we did not find that an individual's mean BOLD activation correlated with their mean performance on the task, there was a significant relationship between the overall level of BOLD activation in an individual's M1 ROI and the likelihood of observing a non-zero slope in the BOLD response as target size changed. This raises the question of whether there could be a floor effect, with a linear change in activation less likely to be detected due to noise levels if all activation values are low. However, a floor effect solely affecting the patients is unlikely, given the mean BOLD response did not differ between the contralesional/ipsilateral M1s in patients and controls, and a linear relationship was detected in ipsilateral M1 in the healthy control participants. Additionally, the sample size of stroke participants is approximately two-thirds of the sample size of the control group. Combined with the lower overall activation in contralesional/ipsilateral M1, the small sample size may prevent us from detecting demand-dependent activation in the stroke patient sample, though a direct comparison of the demand slopes for patients and controls suggests that the slopes are significantly different between the two groups.

Another possible consideration for explaining differences in the demand dependence of contra- and ipsilesional M1 is related to the concept of hemispheric specialization and whether the task was executed with the dominant or non-dominant hand. Here, each M1 contributes differently to the control of both hand movements and motor learning/adaptation [see (61) for a review]. Specifically, in this model, the left hemisphere provides predictive control mechanisms specifying aspects such as movement direction and curvature, whereas the right hemisphere specializes in impedance control to improve final position accuracy. While this is an important model for understanding motor control, in the current analysis we could not consider hemispheric specialization with respect to the left and right sides because of the mixed handedness of our patients (see Table 1). Relating differences in the demand-dependent BOLD response of contralesional M1 to hemispheric specialization must be addressed in a future study.

Sensorimotor brain areas beyond M1 are active in this and other motor tasks and contribute to post-stroke motor control and functional recovery (17, 62–65). However, given that M1 is a common target of various rehabilitation treatment approaches (28, 66), our hypothesis about the role of contralesional M1 in supporting hand motor function after stroke is specific to M1. We have limited our analyses here to individually localized functional ROIs that largely encompass the M1 hand area in both hemispheres.

Here we show that, similar to healthy age-matched controls, patients with subacute ischemic stroke performing a skilled hand motor task show demand-dependent accuracy and movement times and a linear increase in their BOLD response in ipsilesional M1. In contrast to the controls, this relationship was weaker in the contralesional M1. Longitudinal work assessing changes in demand-dependent activity over time and its relationship to the recovery of function in patients with stroke is needed to further clarify if reorganizational changes in M1 are driving the observed weaker relationship between the contralesional BOLD response and the demand on precision. However, this is a possible explanation given the evidence from the rodent stroke models (30–36) and non-human primates (67–69). It also remains to be determined whether this is also seen in patients with greater injury to M1 and CST. The findings described above suggest that some of the BOLD response observed in contralesional M1 is related to the demand of the motor task being performed. Therefore, some of the reported abnormal bilateral M1 activations in patients with stroke (13) could be a response to the relatively higher demand of the task when compared to healthy controls. In future studies, care should be taken to quantify the demand of a motor task to account for these differences. An improved understanding of the role of contralesional M1 in supporting compromised hand function is critical for the design and selection of safe and evidence-based treatment approaches in neurorehabilitation.

## Data Availability Statement

The raw data supporting the conclusions of this article will be made available by the authors, without undue reservation.

## Ethics Statement

The studies involving human participants were reviewed and approved by Emory University Institutional Review Board. The patients/participants provided their written informed consent to participate in this study.

## Author Contributions

KR, CB, and MH conceived and designed research. KR, CB, DB, AC, and IV collected the data. FN, SB, IV, and CB selected and recruited stroke patients for potential participation. KR, DB, MH, SR, and JT analyzed data. KR, CB, and DB interpreted results of experiments. KR prepared figures. KR and CB drafted the manuscript. KR, DB, MH, SB, FN, and CB edited and revised manuscript. KR, DB, AC, IV, SR, JT, SB, FN, MH, and CB approved final version of manuscript. All authors have read and agreed to the published version of the manuscript.

## Funding

This work was supported by the National Institutes of Neurological Diseases and Stroke and the National Institutes of Child Development and Health at the National Institutes of Health, Bethesda, MD, USA (R01NS090677). DB received support from the Emory University NIH/NINDST32 training and translational research in neurology program (T32NS007480), Georgia StrokeNet, and the American Heart Association (18POST34060183). The content is solely the responsibility of the authors and does not necessarily represent the official views of the National Institutes of Health.

## Conflict of Interest

The authors declare that the research was conducted in the absence of any commercial or financial relationships that could be construed as a potential conflict of interest.

## Publisher's Note

All claims expressed in this article are solely those of the authors and do not necessarily represent those of their affiliated organizations, or those of the publisher, the editors and the reviewers. Any product that may be evaluated in this article, or claim that may be made by its manufacturer, is not guaranteed or endorsed by the publisher.
